# A case of anal canal carcinoma with pagetoid spread that was curatively resected by multiple endoscopic and surgical treatments

**DOI:** 10.1002/deo2.340

**Published:** 2024-02-09

**Authors:** Koichi Furuta, Yoshiaki Kimoto, Yuki Kano, Takashi Sakuno, Kohei Ono, Yohei Minato, Kentaro Nakajima, Sakiko Miura, Teppei Morikawa, Ken Ohata

**Affiliations:** ^1^ Department of Gastrointestinal Endoscopy NTT Medical Center Tokyo Tokyo Japan; ^2^ Department of Surgery NTT Medical Center Tokyo Tokyo Japan; ^3^ Department of Histopathology NTT Medical Center Tokyo Tokyo Japan

**Keywords:** anal canal carcinoma, endoscopic submucosal dissection, extra‐mammary Paget's disease, mapping biopsy, pagetoid spread

## Abstract

A 57‐year‐old woman with no significant medical history was referred after a colonoscopy for abdominal distension, which revealed a tumor in the lower rectum. Pre‐operative colonoscopy showed the tumor was 12 mm in size, located from the anorectal junction to beyond the dentate line, and was diagnosed as high‐grade intramucosal neoplasia or shallow submucosal invasive cancer. Endoscopic submucosal dissection was performed, and the lesion was resected en bloc. Pathological examination revealed moderately differentiated tubular adenocarcinoma with tubulovillous adenoma. The stratified squamous epithelium adjacent to the anal side of the lesion showed pagetoid spread of atypical cells with positive horizontal margins. We referred her to a surgeon for radical treatment. The mucosa surrounding the endoscopic submucosal dissection scar was normal on narrow‐band imaging magnification. We marked its oral side endoscopically as the resected boundary. Transanal local excision was performed. The horizontal margins were positive because atypical cells had spread into the stratified squamous epithelium of the anorectal side of the lesion. The patient was followed on an outpatient basis. Sixty days postoperatively, residual tumor growth was observed. The second local resection was performed after mapping biopsy. All resection margins were negative, there was no lymphovascular invasion. One year after surgery, no recurrence was observed. Regarding endoscopic findings, there are no reports of endoscopic findings of the rectal mucosa, or the squamous epithelium of the anus of pagetoid spread. Here, we report a review of perianal Paget's Disease that resulted in difficulties in borderline diagnosis of pagetoid spread, resulting in multiple therapeutic interventions.

## INTRODUCTION

Extra‐mammary Paget's Disease (EMPD) has been reported in several extramammary sites, such as the axilla, thighs, groin, perineum, scrotum, vulva, and perianal area.^1^, ^2^ If it presents in the perianal area, it is referred to as perianal Paget's Disease (PPD).^3^ PPD often shows pagetoid spread (PS), a pattern of tumor spread into neighboring organs around the epidermis due to intraepithelial progression.^4^ However, this PS is difficult to visualize endoscopically, and there have been no detailed reports on the endoscopic findings of PPD or the progress of multiple endoscopic treatments. Here, we report a review of PPD that resulted in multiple therapeutic interventions, including detailed endoscopic findings.

## CASE REPORT

**FIGURE 1:**
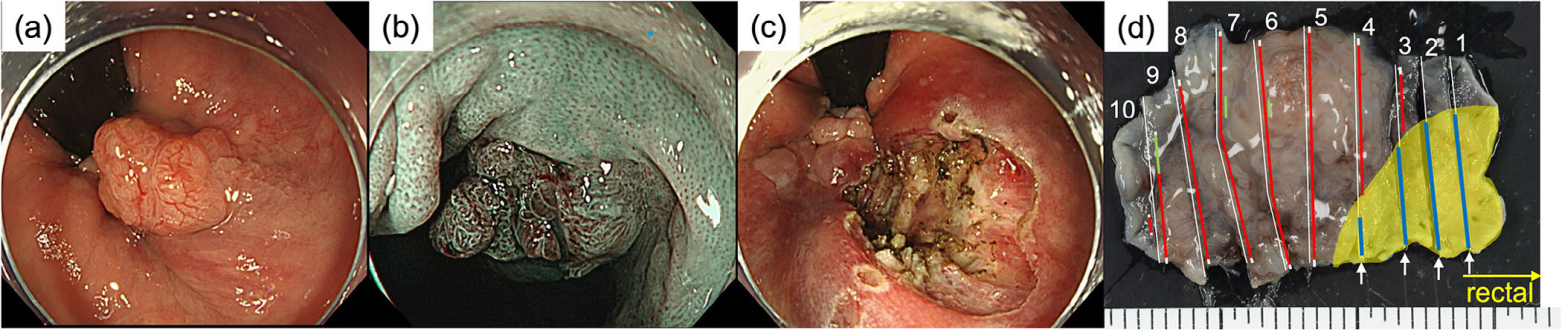
(a) Anal canal tumor was located from the anorectal junction to beyond the dentate line. (b) The surface structure and microvessels were evenly well‐organized with narrow‐band imaging magnification. (c) Resected ulcers had no endoscopically recognizable tumor remnants. (d) The mapping image of postoperative pathology. The depth was intramucosal, vertical dissection was negative, and there was no lymphovascular invasion. However, the stratified squamous epithelium adjacent to the anorectal side of the lesion had positive horizontal margins. (red line: intramucosal tumors, green line: tubulovillous adenoma [White lines are appended to both ends of the mapping in areas where the horizontal margins are negative], yellow area: stratified squamous epithelium, blue line: Pagetoid spread, white arrow: positive horizontal margins)

**FIGURE 2:**
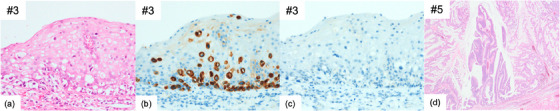
Hematoxylin and eosin staining (a) Specimen #3 from Figure 1d exhibits stratified squamous epithelium with Pagetoid spread of atypical cells (×40). Immunohistological staining of atypical cells in stratified squamous epithelium for (b) CK20 was positive (×40), while (c) GCDFP15 was negative (×40). Hematoxylin and eosin staining (d) rectal region #5 from Figure 1d (×4). The final pathological diagnosis was moderately differentiated tubular adenocarcinoma (tub2>tub1) with tubulovillous adenoma, type 0‐Is, 25 × 15 mm/25 × 18 mm, pTis (depth M), Ly0, V0, pHM1, and pVM0.

A 57‐year‐old woman with no significant medical history was referred after a colonoscopy for abdominal distension, which revealed a tumor in the lower rectum. Pre‐operative colonoscopy revealed the tumor was located from the anorectal junction to beyond the dentate line. The tumor was 12 mm in size and sessile type. With narrow‐band imaging (NBI) magnification, the surface pattern showed irregular, and the vascular pattern showed variable caliber and irregular distribution. Dotted blood vessels without atypia were observed in the squamous epithelium adjacent to the anal side of the lesion, and we concluded that there was no invasion into the squamous epithelium (Figure 1a,b). Therefore, we recognized the border between the reddish elevation and the surrounding squamous epithelium as the demarcation line. We diagnosed the lesion as high‐grade intramucosal neoplasia or shallow submucosal invasive cancer based on these endoscopic findings. As the lesion was located on the anorectal side beyond the dentate line of the surgical anal canal, we concluded that endoscopic mucosal resection was not feasible and planned to perform endoscopic submucosal dissection (ESD). ESD was performed, and the tumor was resected en bloc without adverse events, including squamous epithelium on the anorectal side below the dentate line as margin (Figure 1c). Pathological examination revealed that the lesion was a moderately differentiated tubular adenocarcinoma with well‐differentiated components and tubulovillous adenoma (Figure 2a). The depth was intramucosal, the vertical margin was negative, and there was no lymphovascular invasion. However, atypical cells spread into the stratified squamous epithelium adjacent to the anorectal side of the lesion and horizontal margins were positive (Figure 1d).

**FIGURE 3:**
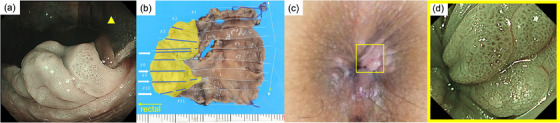
(a) Endoscopic forward view with anoscope observing the skin on the anorectal side rather than the endoscopic submucosal dissection scar. Tumor boundaries could not be recognized by narrow‐band imaging observation. (Yellow triangle: anoscope). (b) The mapping image of postoperative pathology. Pathologic examination showed no residual tumor in the rectal mucosa of the resected specimen. However, atypical cells spread into the stratified squamous epithelium on the anorectal side and horizontal margins were positive. (yellow area: stratified squamous epithelium, blue line: Pagetoid spread, white arrow: positive horizontal margins). (c) Yellow square: The pink elevated area is an enlarged residual lesion that has appeared in the perianal area. (Sixty days after transanal local excision). (d) NBI magnification of the enlarged residual tumor. The same area as the yellow square in Figure 3(c). Dotted blood vessels with no atypia were observed on the surface of the recurrent tumor.

**FIGURE 4:**
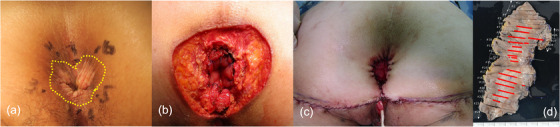
(a–c) Macroscopic view of the perianal skin. (a) We performed a mapping biopsy of 1 cm separate concentric areas around the anal erosion. (Yellow dotted line: extent of tumor spread as estimated from the postoperative specimen.). (b) After local resection of the perianal skin lesion. (c) After reconstruction with myocutaneous valve. (d) The mapping image of postoperative pathology. Pathologic diagnosis showed residual adenocarcinoma with pagetoid spread, negative margins, and no lymphovascular invasion (red line: Pagetoid spread of atypical cells).

In addition, immunohistological staining of atypical cells in the stratified squamous epithelium was positive for CK20, whereas GCDFP15 was negative (Figure 2c,d). The outcome revealed the manifestation of PS of colorectal malignancy, and the primary EMPD diagnosis was negated. We chose transanal local excision for radical resection to preserve anal function because the depth of the rectal side was intramucosal. We conducted endoscopic observations to determine the extent of resection, and by performing NBI magnification (Figure 3a), we determined the mucosal tissue encompassing the ESD scar to be normal. The resection area was wider, 4×3 cm from the resection margin on the oral side to secure the margins. The oral side of the specimen (rectal mucosa) had no residual tumor, but the anal side (stratified squamous epithelium) had a positive horizontal margin because atypical cells spread into that (Figure 3b). After discussion with the surgeon, we decided that surgical resection was not a reasonable option for the lesion that was difficult to visualize endoscopically and decided on endoscopic follow‐up. Sixty days after surgery, a gross lesion appeared in the perianal area, and it was determined that the residual tumor had grown (Figure 3c). Dotted blood vessels were observed on the surface of the recurrent tumor, but there was no atypia (Figure 3d). So, we determined the area of the anal skin resection with a mapping biopsy (Figure 4a). A second local resection was performed, which included rectal mucosa and anal skin excision, bilateral inguinal lymph node dissection, and myocutaneous valve reconstruction (Figure 4b,c). Pathologic diagnosis revealed residual adenocarcinoma with PS, negative margins, and no lymphovascular invasion (Figure 4d). We determined that R0 resection was achieved. One year after surgery, no recurrence was observed.

## DISCUSSION

PS is the spreading of carcinoma adjacent to the squamous epithelium and has histologic features similar to Paget cells.^4^ PS and primary EMPD are clinically and pathologically very similar. Immunohistochemistry has usually been used to differentiate between primary EMPD and PS. In many cases of PS, GCDFP15 is negative, and CK20 is positive,^5^, ^6^, ^7^, ^8^ so this case was also diagnosed as PS. Primary EMPD is mostly intraepidermal carcinoma and has a good outcome.

On the other hand, PS is usually associated with an underlying malignant anorectal tumor and has a relatively poor prognosis^9^ with a high risk of local recurrence,^3^ so that abdominoperineal resection is usually performed, and anal function is not preserved.^10^ Here, we experienced a case in which the tumor border on the rectal mucosa was recognized by NBI magnification, but the border on the squamous epithelial side could not be diagnosed endoscopically. To date, there have been no reports that have examined in detail the endoscopic findings of squamous epithelium in the anal skin in PS. Therefore, it was suggested that the boundaries of the squamous epithelium of the anal skin beyond the dentate line may not be recognizable endoscopically. In this case, although the stratified squamous epithelium adjacent to the anorectal side of the lesion of ESD had positive horizontal margins, the tumor cells existed only in the mucosal layer. Therefore, we concluded that abdominoperineal resection was unnecessary for radical treatment and that local excision would be curative. The rectal border of the lesion could be recognized endoscopically, so the resection was minimal. However, the anorectal border could not be recognized even with NBI magnification, requiring multiple surgical treatments. A mapping biopsy is usually performed when the anorectal lesion is macroscopically visible,^2^ as in the second local excision in this case. If the tumor boundary cannot be recognized grossly or endoscopically, as in this case, the extent of resection should be determined using intraoperative consultation, for example. In conclusion, we report a review of PPD that resulted in difficulties in borderline diagnosis of PS, resulting in multiple therapeutic interventions.

## CONFLICT OF INTEREST STATEMENT

None.

## ETHICS STATEMENT

All procedures followed were performed in accordance with the ethical standards laid down in the Declaration of Helsinki and its later amendments.
